# Endometriosis and physical exercises: a systematic review

**DOI:** 10.1186/1477-7827-12-4

**Published:** 2014-01-06

**Authors:** Camila M Bonocher, Mary L Montenegro, Julio C Rosa e Silva, Rui A Ferriani, Juliana Meola

**Affiliations:** 1 Department of Gynecology and Obstetrics, Faculty of Medicine of Ribeirão Preto, University of São Paulo, Ribeirão Preto 14049-900, Brazil

**Keywords:** Endometriosis, Physical exercise, Life style

## Abstract

Regular physical exercise seems to have protective effects against diseases that involve inflammatory processes since it induces an increase in the systemic levels of cytokines with anti-inflammatory and antioxidant properties and also acts by reducing estrogen levels. Evidence has suggested that the symptoms associated with endometriosis result from a local inflammatory peritoneal reaction caused by ectopic endometrial implants. Thus, the objective of the present review was to assess the relationship between physical exercise and the prevalence and/or improvement of the symptoms associated with endometriosis. To this end, data available in PubMed (1985–2012) were surveyed using the terms “endometriosis and physical exercises”, “endometriosis and life style and physical exercises” in the English language literature. Only 6 of the 935 articles detected were included in the study. These studies tried establish a possible relationship between the practice of physical exercise and the prevalence of endometriosis. The data available are inconclusive regarding the benefits of physical exercise as a risk factor for the disease and no data exist about the potential impact of exercise on the course of the endometriosis. In addition, randomized studies are necessary.

## Background

Endometriosis is a benign estrogen-dependent gynecological disease that affects 10 to 15% of women of reproductive age and is characterized by the growth of endometrial tissue outside the uterine cavity [1]. The most common site of endometriotic implants is the pelvic cavity, especially the pelvic and ovarian peritoneum, but implants can also be found in the posterior cul-de-sac, rectovaginal septum, intestine, and bladder. Lesions in the pericardium, pleura, liver, kidney, bladder, brain, lower limbs, and nasal cavity have also been reported [2]. Some symptoms are characteristic of endometriosis, such as dysmenorrhea, dyspareunia, non-cyclic pelvic pain, and infertility [3]. The prevalence of endometriosis ranges from 2 to 22% in reproductive aged women and may reach 40 to 60% among women with dysmenorrhea [4]. In addition, about 25 to 50% of infertile women have endometriosis [5].

Evidence suggested that these symptoms of the disease result from a local inflammatory peritoneal reaction caused by the ectopic endometrial implants [6], which undergo cyclic bleeding [7]. Oxidative stress seems to be involved in the physiopathology of endometriosis since reactive oxygen species appear to be increased in the peritoneal fluid of women with endometriosis [8]. These changes contribute to the development and maintenance of the inflammatory process associated with endometriosis. On the other hand, regular physical exercise seems to have protective effects against diseases that involve inflammatory processes since it induces an increase in the systemic levels of cytokines with anti-inflammatory properties [9]. In addition, regular physical exercise is associated with a cumulative effect of reduction of menstrual flow, of ovarian stimulation and of the action of estrogen [10].

On this basis, it is possible that the practice of physical exercise has beneficial effects on endometriosis. Thus, the objective of the present review was to survey the literature for data that may support the effects of physical exercise on women with endometriosis in terms of prevalence, and possible therapeutic effects of physical exercises. This review also tried clarify if the pelvic pain caused by the disease can somehow impair the practice of physical exercise in women with endometriosis.

## Methods

This study is a systematic review. It was based on the survey of data available in PubMed (1985 to September 2012). The terms investigated were “endometriosis and physical exercises”, “endometriosis and life style and physical exercises” and “endometriosis and risk factor”. Three reviewers analyzed the data in an independent manner and only studies having at least one of the following characteristics were considered: observational or experimental, analytical or descriptive studies of the association between physical exercise and endometriosis diagnosed by laparoscopy. Review and opinion studies were excluded as well as non-English manuscripts.

## Results

The survey of the chosen terms revealed 935 articles, only 6 of which were considered for review (Table 1) by satisfying the inclusion criteria established, i.e. direct link between the practice of physical exercise and the prevalence of endometriosis. Six studies were fully analyzed and the results are not comparable with each other as described in Table 1.

**Table 1:** Data extracted from the articles selected for a more detailed analysis

**Reference**	**Objective**	**Design**	**Materials**	**Sample size**	**Physical exercise/endometriosis association**
**Cramer DW et al. **[11]	Comparison of menstrual and constitutional factors in women with and without endometriosis	Retrospective comparative	Questionnaire focused on demographic, menstrual, reproductive and exercise history	268 cases of endometriosis and 3794 controls	A lower risk for women who practiced regular exercise a minimum of 2 h/week
**Han M et al. **[12]	Provide information about the prophylaxis of the endometriosis	Case–control	Questionnaire focused on demographic, menstrual, reproductive and exercise history	203 cases of endometriosis and 406 controls	Avoidance of strenuous exercise during the menstruation is a preventive factor for endometriosis
**Signorello LB et al. **[13]	Identify risk factors for endometriosis	Case–control	Questionnaire focused on menstrual, marital, reproductive and exercise history	50 cases of endometriosis and controls: 47 infertile/89 fertile	Practice 2 to 4 hours per week of regular exercise decreases 65% chance of endometriosis
**Dhillon PK and Holt VL. **[14]	Evaluate the risk of endometrioma associated to physical activity	Case–control	Questionnaire focused on menstrual, marital, reproductive and exercise history	77 cases of endometrioma and 735controls	Frequent and high-intensity activity during 2 years reduced 76% of endometrioma risk
**Vitonis AF et al. **[15]	Clarify the relationship between physical activity and endometriosis	Prospective cohort study	Questionnaire focused on demographic, biologic and lifestyle risk factors	2703 cases of endometriosis and controls: 1857 fertile/846 infertile	Aerobic exercise had a protective effect in women with confirmed endometriosis
**Koppan A et al. **[16]	Identify factors that influence on quality of life and pain scores in women with endometriosis	Case–control	Questionnaire focused on age, marital, education, reproductive, medical and exercise history	81 cases of endometriosis: 31 regular exercise/50 no exercise	Taking painkillers is less effective in women with endometriosis that practice regular daily sport activities

The first epidemiological study relating physical exercise and endometriosis was published in 1986. Cramer et al. [11] compared the characteristics of the menstrual cycle and of constitutional factors in 268 white women with primary infertility due to endometriosis with laparoscopic confirmation and in a control group without laparoscopic exclusion of the disease. The study demonstrated that women who exercised regularly before the beginning of the study had a significantly lower risk for endometriosis compared to women who did not exercise. On this basis, physical exercise had a protective effect limited to women who started exercising before 26 years of age, at least two hours per week.

The type of associated exercise was physical conditioning such as running and gymnastics. Approximately 12% of the patients and controls reported less pain and change in the menstrual cycle after the beginning of the exercise program. The authors did not point if in women with endometriosis, the pelvic pain was a limiting factor for exercise practice. It should be remembered that the constitutional factors taken into account in this study were those that might affect the levels of endogenous estrogen such as smoking habit, body habits and physical exercise.

Han et al. [12] demonstrated that women with early menarche (≤12 years) and a menstrual period of 8 days or more were at higher risk to develop endometriosis, and that the risk tended to increase in association with primary dysmenorrhea, intense physical activity during the menstrual period and allergic diathesis. This study also showed that avoidance of strenuous exercise during menstruation was identified as a preventive factor of endometriosis and that because the dysmenorrhea some women chose not to exercise during their periods.

In a case–control study, Signorello et al. [13] related the risk of endometriosis to other less studied factors such as anthropometric variables (height, weight and BMI), physical exercise, smoking, and alcohol consumption. The authors observed an inverse association between vigorous physical exercises and endometriosis, although without statistical significance. The authors also pointed that is possible that the protective effect of exercise in patients with endometriosis result from the fact that women who suffer from endometriosis do not feel well enough to practice exercise. To try decrease the bias, they separate the groups by cases and control that practiced exercise. Women who exercised 4 hours per week or more reduced by 65% the risk of endometriosis compared to women who exercised less than 4 hours a week.

Dhillon and Holt [14] conducted a case–control study about the risk of endometrioma associated with recreational physical activity. The intensity of physical activity was determined based on a metabolic equivalent (MET) using the Compendium of Physical Activities and not simply on a subjective evaluation of what would be “vigorous”, “regular” or “weak” activity. A significant 76% reduction of the risk to develop an endometrioma was observed in patients who exercised frequently (3 times a week or more, 30 minutes or more per episode and 10 months or more per year/2 years) and at high intensity (6.0 METs or more – running, bicycle riding and playing tennis). A reduction of the risk was not observed in patients who practiced exercise of low intensity (less than 4.0 METs – golf, bowling and light walking).

The MET was also used in a study by Vitonis et al. [15]. In a large prospective cohort (Nurse’s Health Study II), these investigators observed that more active women (≥42 MET hours/week) had an 11% reduction of the risk for endometriosis. Considering the physical activity performed 4 years before the diagnosis of endometriosis, there was a 90% reduction of the risk for the disease among patients who exercised 42 MET hours/week compared to those who exercised only 3 MET hours/week. However, there was no relation between a high level of physical activity and endometriosis reported six years before the diagnosis of the disease. There was also an association between inactivity and patients with endometriosis confirmed by laparoscopy. The study revealed a small association between physical activity and the rates of endometriosis confirmed by laparoscopy, and reported that the same protective effect was observed for aerobic exercises among the various types of activity, although the effect of the other activities was small. This study also draws attention to the possibility that pain can influence negatively the practice of physical exercise in women with endometriosis.

Koppan et al. [16] assessed individual factors that influenced the quality of life and the intensity of pain of patients with endometriosis. Their results demonstrated that the use of analgesics could be less effective in patients with endometriosis who exercised regularly. In this study, the authors did not point if in women with endometriosis, the pelvic pain was a limiting factor for exercising.

## Discussion

The protective effects of regular physical exercise have been widely described in the treatment of diseases involving inflammatory processes such as type 2 diabetes and colon and breast cancer [17,18]. Physical activity increases the systemic levels of various cytokines with anti-inflammatory properties [19]. Striated muscle has been recently identified as an endocrine organ, which, through contraction, stimulates the production and release of myocytokines, which may influence and change the metabolism and the production of cytokines in tissues and organs [20,21].

Evidence suggests that the symptoms associated with endometriosis result from a local peritoneal inflammatory reaction caused by the ectopic endometrial implants. Adhesion of ectopic tissue to the peritoneum is believed to occur by means of the integrins, heterodimeric transmembrane receptors of cell adhesion [22], and this adhesion is thought to occur due to disequilibrium between enzymes that degrade the components of the extracellular matrix such as the metalloproteinases (MMPs) and their endogenous regulators, i.e., tissue inhibitors of metalloproteinases (TIMPS). After this peritoneal invasion, the viability of the endometriotic focus may be maintained by local vascular angiogenesis, the latter being stimulated by vascular endothelial growth factor (VEGF) and cytokines. VEGF may be produced and secreted into the peritoneal fluid by infiltrated macrophages, promoting endothelial growth, increased vascular permeability and modulation of the secretion of proteolytic enzyme related to angiogenesis [23,24]. Other studies have demonstrated an association between endometriosis and oxidative stress, which has been pointed out as a factor potentially involved in the physiopathology of endometriosis [8]. Reactive oxygen species seem to be increased in the peritoneal fluid of women with endometriosis. Changes in the expression of enzymes involved in the defense against oxidative stress have also been observed [25,26]. Thus, this evidence strongly suggests the involvement of an inflammatory process in endometriosis [27,28].

Carpenter et al. [29] reported the beneficial effect of physical exercise on the reduction of testosterone levels and of androgenic symptoms in patients treated with Danazol. In another study reporting that physical exercise mitigates the possible adverse effects of medications, Bergström et al. [30] stated that physical exercise is a supporting factor in the recovery of bone density in women treated with GnRH.

Analysis of available literature data show that there are no controlled and randomized studies identifying whether physical exercise prevents the occurrence or progression of the endometriosis and how and to what extent physical exercise could be beneficial for women with endometriosis. The few existing studies are of an observational type, with little or no statistical significance, but that indicate an inverse relationship between the practice of physical exercise and the risk of endometriosis. These studies also drawing attention to the possibility that conclusions about non-protective effect of exercise in women with endometriosis can be due the discomfort experienced, what prevent the practice of physical exercise.

Therefore, with the literature available it is not possible to point out the real role of physical exercise in endometriosis. Thus, until now we only have speculations about this topic. In this respect, we believe that studies well controlled, using validated instruments for evaluation and follow up, well- defined study groups and well established exercise protocol can demonstrate the real role of physical exercise on treatment of endometriosis. On this basis, experimental models of endometriosis would be justified because they would permit the characterization of the time course of the disease in order to elucidate whether physical exercise is indeed able to interfere with the development of the endometriosis injury. In addition, it would be possible to determine what intensity would be necessary for physical exercise to be used in both a preventive and curative manner regarding the disease.

## Conclusions

Literature available is inconclusive regarding the benefits of physical exercise for women with endometriosis. In this sense, controlled and randomized studies are necessary to bridge the literature gap.

## Competing interests

The authors declare that they have no competing interests.

## Authors’ contributions

MLM conceived the idea; CM and RAF did the literature survey; CM, MLM, JCRS, RAF and JM read and analyze independently the selected manuscripts; CM and MLM drafted the manuscript; MLM edited the final document and answered to reviewers. All authors read and approved the final manuscript.
